# Hawk Eyes II: Diurnal Raptors Differ in Head Movement Strategies When Scanning from Perches

**DOI:** 10.1371/journal.pone.0012169

**Published:** 2010-09-22

**Authors:** Colleen T. O'Rourke, Todd Pitlik, Melissa Hoover, Esteban Fernández-Juricic

**Affiliations:** 1 Department of Biological Sciences. California State University Long Beach, Long Beach, California, United States of America; 2 Department of Agriculture - Animal and Plant Health Inspection Service - Wildlife Services, Los Angeles, California, United States of America; 3 Department of Biological Sciences, Purdue University, West Lafayette, Indiana, United States of America; Lund University, Sweden

## Abstract

**Background:**

Relatively little is known about the degree of inter-specific variability in visual scanning strategies in species with laterally placed eyes (e.g., birds). This is relevant because many species detect prey while perching; therefore, head movement behavior may be an indicator of prey detection rate, a central parameter in foraging models. We studied head movement strategies in three diurnal raptors belonging to the Accipitridae and Falconidae families.

**Methodology/Principal Findings:**

We used behavioral recording of individuals under field and captive conditions to calculate the rate of two types of head movements and the interval between consecutive head movements. Cooper's Hawks had the highest rate of regular head movements, which can facilitate tracking prey items in the visually cluttered environment they inhabit (e.g., forested habitats). On the other hand, Red-tailed Hawks showed long intervals between consecutive head movements, which is consistent with prey searching in less visually obstructed environments (e.g., open habitats) and with detecting prey movement from a distance with their central foveae. Finally, American Kestrels have the highest rates of translational head movements (vertical or frontal displacements of the head keeping the bill in the same direction), which have been associated with depth perception through motion parallax. Higher translational head movement rates may be a strategy to compensate for the reduced degree of eye movement of this species.

**Conclusions:**

Cooper's Hawks, Red-tailed Hawks, and American Kestrels use both regular and translational head movements, but to different extents. We conclude that these diurnal raptors have species-specific strategies to gather visual information while perching. These strategies may optimize prey search and detection with different visual systems in habitat types with different degrees of visual obstruction.

## Introduction

Establishing what animals are looking at has intrigued zoologists [1]–[4], because it can help understand not only the mechanisms of visual information gathering (i.e., detection of color, motion, size, etc.), but also the processes behind visual attention (i.e., object recognition, reduction in visual uncertainty, etc.) [5]. Additionally, measuring the targets of visual attention can have important methodological applications for the study of animal communication, social interactions, food search, mate choice, and anti-predator behavior in multiple taxa [6]–[8].

However, determining targets of visual attention can be challenging because visual systems vary between taxa. For instance, some species have their orbits frontally placed in the skull (e.g., primates), while others have them laterally placed (e.g., birds, lizards). In species with frontally placed eyes, visual targets are associated with the frontal orientation of the head [9]. In species with laterally placed eyes, visual targets can be at both sides of the head simultaneously, rather than at the front [8]. For example, birds view distant objects in front of them by turning their heads sideways [10]–[12]. For species with laterally placed eyes, other indictors of monitoring behavior can be used, such as the rate of head movements or the interval between consecutive head movements [11], [12].

Variations in the rate of head movements in birds have been associated with different predator scanning [13] and foraging [14], [15] strategies, monitoring the presence of kin in groups [16], and prey searching under different ambient light conditions [12]. Migratory birds even modify the orientation of their heads to track the magnetic field [17]. Furthermore, some head movement patterns are used with specific types of body movements; species with large stride length tend to head-bob (e.g., Gray Heron *Ardea cinerea*), whereas species with short strides do not head-bob (e.g., Pintails *Anas acuta*) [18].

Patterns of variation in head movement behavior can shed light into the visual mechanisms used to obtain information from specific objects in the environment. For instance, hens (*Gallus domesticus*) tend to move their heads sideways at a faster pace when exposed to novel visual stimuli, suggesting that individuals move the image from the right to the left eye to visually explore objects [11]. However, relatively little is known about the degree of inter-specific variability in head movement strategies in species with laterally placed eyes when stationary individuals scan the environment. This is relevant because many species detect prey while perching (e.g., sit-and-wait predators [19]); thus, head movement behavior may be an indicator of prey detection rate, a central parameter in foraging models [20], [21].

Birds of prey (hereafter: “raptors”) constitute an interesting study system to assess inter-specific variability in head movement strategies because they rely on vision to detect and capture prey, hunt different prey types, and inhabit environments with different degree of visual complexity (e.g., open vs. closed habitats). The visual system of diurnal raptors can be generally characterized as having large eyes, high acuity, two visual foveae, relatively narrow binocular visual fields, and eye movement amplitude that varies between species [10], [22], [23]. The fovea is a displacement of the inner layers of the retina that form a depression with higher density of retinal ganglion cells in the perifoveal area [24], [25]. The central fovea that projects sideways into the visual field tends to have higher acuity than the temporal fovea that projects frontally [25], [26]. Despite this general pattern, a recent study [23] found between-species differences in visual field configuration and degree of eye movement in three diurnal raptors: Red-tailed Hawk *Buteo jamaicensis*, Cooper's Hawk *Accipiter cooperi*, and American Kestrel *Falco sparverius*. The goal of this study was to assess between-species variation in head movement strategies in these three raptors.

Red-tailed Hawks are sit-and-wait predators that prey upon mammals, reptiles, and birds, which are generally spotted from exposed perches in open habitats [27]. They have large blind areas (82°) above and at the rear of their heads, medium-sized lateral fields (122°), and an intermediate degree of eye movement (5°) [23]. Cooper's Hawks are active-ambushing predators that inhabit closed habitats and prey upon mammals and birds [28]. Cooper's Hawks' have the widest binocular fields (39°) of the three species, large lateral fields (132°), small blind areas (60°), and a high degree of eye movement (8°) [23]. American Kestrels prey upon small mammals and large insects in open habitats from perches or by hovering and stooping down onto prey [29]. They have large lateral areas (130°), medium-sized blind areas (68°), and a low degree of eye movement (1°) [23].

Despite variations in the size of the lateral fields, all three species use their lateral vision to explore objects [10], as this is the sector of the visual field subtended by the fovea [26]. We predicted that head movement rates would be higher in Cooper's Hawks than in the other two species as a result of the relative distance to visual obstructions in the environment due to vegetation structure. Cooper's Hawks search for prey in closed habitats, which would decrease the ability of individuals to track prey items by obstructing the line of sight close to them [15]. Conversely, we predicted that Red-tailed Hawks and American Kestrels would have lower head movement rates, hence lengthening the interval between consecutive head movements, as they search for prey in visually open habitats, and thus the visual obstruction by less complex vegetation (e.g., grassy areas) is farther away.

Another component that enhances prey detection is motion parallax [30], [31], which provides depth cues though changes in the relative position of objects at different distances caused by the movement of the observer [32]. Specific types of head movements (e.g., vertical or frontal displacements of the head keeping the bill in the same direction) have been associated with motion parallax cues [31], [33], [34]. However, recent studies found that some types of eye movements (e.g., smooth tracking) may bear an even more important role than head movements in providing motion parallax information [35], and that the amplitude of eye movement is related to the perceived depth [36]. This suggests that both head and eye movements may be implicated in motion parallax at different viewing distances [36], [37]. Consequently, we predicted that American Kestrels would compensate for their reduced degree of eye movement (as reported in an accompanying study [23]) by showing higher rates of head movements involved in motion parallax than the two hawk species studied.

## Methods

Using video recordings, we measured the rate of two types of head movements: regular (head moves along a single axis and the direction of the eye-bill tip vector follows the head movement [31]) and translational (head moves along a combination of axes in a straight or curved path but without changing the orientation of the eye-bill tip vector [31], [33]). Translational head movements have been suggested to be used to gather depth cues through motion parallax [30], [31], [38]. Additionally, we measured the interval duration between consecutive regular head movements as a proxy of the amount of time a given head position was maintained [11].

We obtained videos of the three species from the Macaulay Library Sound and Video Catalog (http://animalbehaviorarchive.org). This database has been used extensively as a data source for different studies (see http://macaulaylibrary.org/inside/use/research/publications.do). The exact geographic location of the archive videos was not always known. We selected videos taken in habitats characteristic of the three species. We distinguished between open and closed habitats based on the amount of visual obstruction in the environment (e.g., higher in closed habitats) and the relative distance between vegetation and the animals (e.g., longer in open habitats). We were able to assess the habitat type from the background vegetation from most of the videos.

We only used videos of perched individuals, as head movements could not be accurately measured from flying individuals. We selected videos based on overall quality (mean length was 57±5 s), ensuring that head movements could be accurately identified. Videos at the Macaulay Library Sound and Video Catalog are listed with information on the month and location the video was taken, and the observer who took the video. For some videos, not all of this information was available, but as long as the videos differed in at least one of these categories, we assumed they were recorded on different individuals. When multiple videos from the same bird were available, we used only the longest video clip. Videos that showed inter- or intra- specific interactions (e.g., mobbing by smaller birds, intra-specific aggression), preening events, feeding while perching or under extreme weather conditions were not included in the analysis.

To obtain a minimum of 10 videos per species, we recorded additional videos ourselves from locations with similar habitat to those found in the cataloged videos. Recordings were obtained in Los Angeles and Orange Counties (California) between June and August 2008 using a Sony Handycam DCR-HC36. We recorded videos at different times of the day. Overall, sample sizes per species were: Red-tailed Hawk (16, catalog only), Cooper's Hawk (9, catalog; 1, recorded by authors), and American Kestrel (8, catalog; 2, recorded by authors). All our video recording procedures complied with approved protocols to work with these species (CSULB Protocol No. 256).

One of the many shortcomings of field observations is that head movements may have been affected by the visual background animals experienced during the recordings. Therefore, to further characterize the inter-specific differences in scanning behavior with similar visual backgrounds, we recorded videos from individuals of the three species under captive conditions. Recordings were made at the Lindsay Wildlife Museum (Walnut Creek, CA), the Indianapolis Zoo (IN), and three raptor facilities in Indiana (Delphi, Rochester, Redkey). Although the size of the enclosures in which animals were held differed between institutions, focal animals were not blocked by vegetation. Recordings were made on different individuals between May and June 2010 (in the early morning or late afternoon) with a Sony Handycam DCR-HC36. All animals were recorded perching, with the position of the observer varying from 5–15 m from the enclosure. We did not handle any animal while obtaining these videos. We recorded 6 Red-tailed Hawks, 5 American Kestrels, and 3 Cooper's Hawks. Mean video length was 231±42 s.

We recorded regular and translational head movements with JWatcher [39]. Individuals from all three species showed both head movement types while perching with the body still, so that head movement represents a visual scan of the environment rather than a stabilizing movement. We calculated the rate of each type of head movement (changes in head position per min). We also calculated the interval duration between consecutive regular head movements (millisecs) as the average time the head was stationary before performing the next movement [40].

### Statistical analysis

We used MANOVAs to determine the differences between the rate of regular and translational head movements pooling all species and within each species. ANOVAs were used to assess differences among species in the three head movement parameters studied under field and captive conditions: regular head movement rate, translational head movement rate, and interval duration between consecutive regular head movements. Tukey tests were used for pair-wise comparisons. P-levels <0.05 were considered significant. Translational head movement rate (field observations) and intervals between consecutive regular head movements (captive observations) were log (x +1) transformed to meet normality assumptions; however, figures show untransformed values for clarity. We present means (± SE) throughout.

## Results

**Figure 1:**
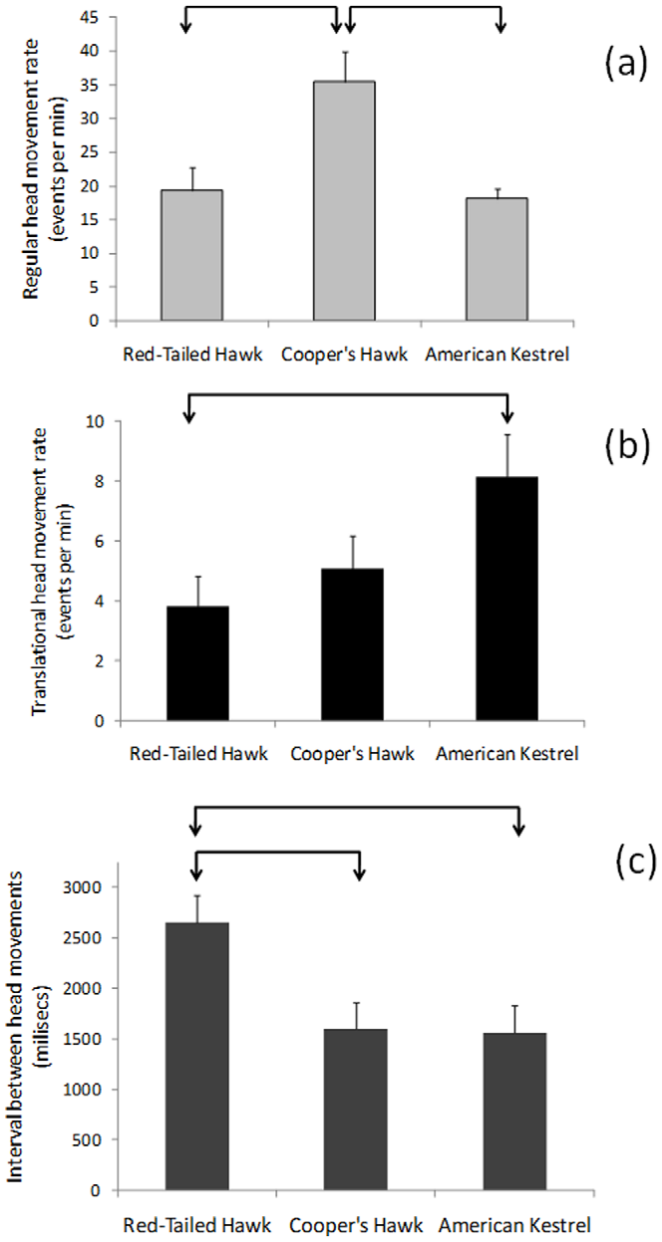
Head movement behavior of three diurnal raptors recorded in field conditions. (a) Regular head movement rates, (b) translational head movement rates, and (c) average duration of the intervals between consecutive regular head movements of Red-tailed Hawks, Cooper's Hawks, and American Kestrels recorded in field conditions. Statistical analyses on translational head movement rates were conducted on log (x +1) transformed values to meet model assumptions; however, the figure shows untransformed values. Arrow-bars represent significant (P<0.05) differences from pair-wise Tukey tests.

Pooling all species, the rate of regular head movements (21.33±2.50) was higher than that of translational head movements (4.67±0.64; F_2,34_ = 60.80, P<0.001). Within each species, we also found that regular head movements were used significantly more frequently than translational head movements (Fig. 1a–b): Red-tailed Hawks (F_2,14_ = 86.77, P<0.001), Cooper's Hawks (F_2,8_ = 76.32, P<0.001), American Kestrels (F_2,8_ = 60.30, P<0.001).

We found significant differences in the rate of regular head movements among species (F_2,33_ = 9.83, P<0.001). Cooper's Hawks had the highest regular head movement rate, which differed significantly from those of Red-tailed Hawks and American Kestrels (Fig. 1a), without significant differences between the latter two species (Fig. 1a).

Translational head movement rates also differed significantly among species (F_2,33_ = 3.79, P = 0.037). American Kestrels had a significantly higher rate of translational head movements than Red-tailed Hawks (Fig. 1b). However, we did not find significant differences between American Kestrels and Cooper's Hawks and between both species of hawks (Fig. 1b).

The interval duration between consecutive regular head movements varied significantly among species (F_2,33_ = 5.34, P = 0.010). Red-tailed Hawks had the longest interval, which differed significantly from Cooper's Hawks and American Kestrels (Fig. 1c). However, we did not find significant differences between Cooper's Hawks and American Kestrels (Fig. 1c).

**Figure 2:**
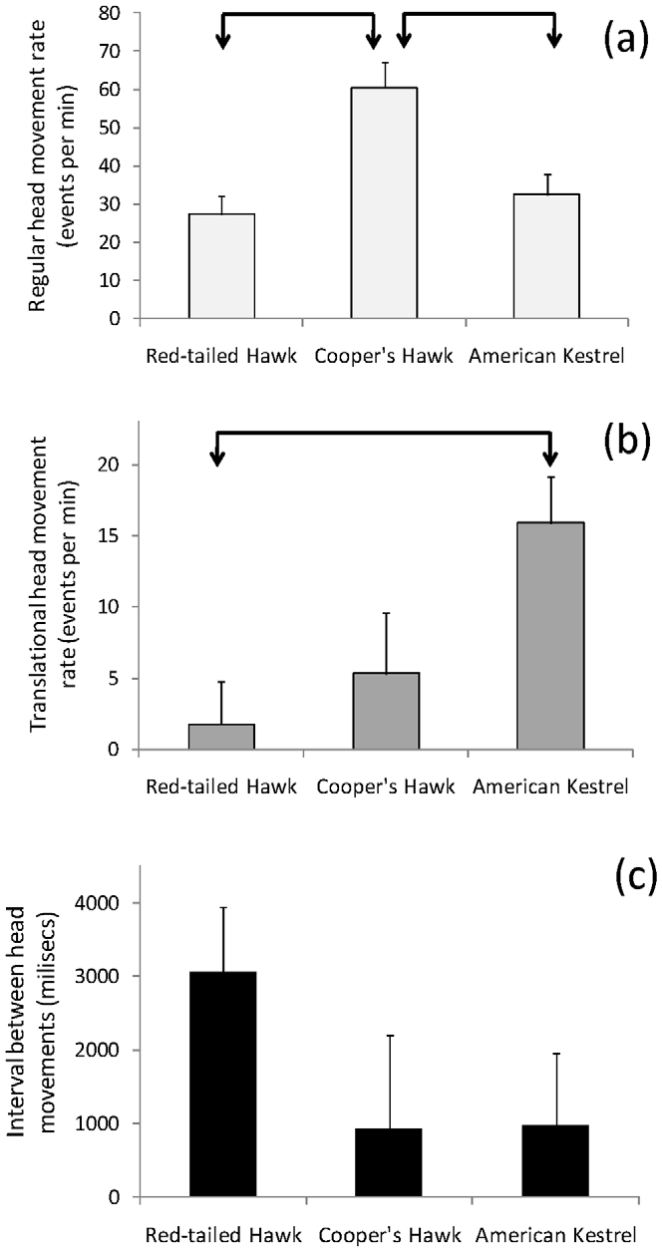
Head movement behavior of three diurnal raptors recorded in captive conditions. (a) Regular head movement rates, (b) translational head movement rates, and (c) average duration of the intervals between consecutive regular head movements of Red-tailed Hawks, Cooper's Hawks, and American Kestrels recorded in captive conditions. Statistical analyses on the intervals between consecutive regular head movements were conducted on log (x +1) transformed values to meet model assumptions; however, the figure shows untransformed values. Arrow-bars represent significant (P<0.05) differences from pair-wise Tukey tests.

Our recording under captive conditions generally corroborated the findings obtained in the field. First, Cooper's Hawks showed the highest rate of regular head movements (F_2,11_ = 8.35, P = 0.006), which differed significantly from those of the other two species (Fig. 2a). Second, American Kestrels had the highest rate of translational head movements (F_2,11_ = 5.25, P = 0.025), which varied significantly from those of Red-tailed Hawks (Fig. 2b). Third, we found a significant difference among species in the interval between consecutive regular head movements (F_2,11_ = 4.48, P = 0.038; Fig. 2c). The trend was for Red-tailed hawks to have the longest intervals between consecutive regular head movements (Fig. 2c); however, the post-doc tests did not reach the significance level (Red-tailed Hawk vs. American Kestrel, P = 0.059; Red-tailed Hawk vs. Cooper's Hawk, P = 0.086). Examples of the head movement strategies of these three species are presented in the Supporting Information: (a) links to field videos from the Macaulay Library Sound and Video Catalog (Text S1) and (b) videos we obtained from each species (Video S1, Video S2, Video S3).

## Discussion

We found between-species differences in head movement rates and duration that are indicative of species-specific scanning strategies. Previous studies have characterized head movements in raptors flying, particularly in pursuit of prey [10], [41], [42]. In general, moving the head while flying appears to be less flexible than when perching, partly due to the aerodynamic drag caused by sideways head movements [10], [41]. Our study quantified different head movement parameters in three perching diurnal raptors (see [34] for non-raptors). Comparatively, Red-tailed Hawks showed long intervals between consecutive regular head movements; Cooper's Hawks, high rates of regular head movements; and American Kestrels, high rates of translational head movements. These scanning strategies may be used in prey searching, and may be particularly relevant for these sit-and-wait and sit-and-pursue predators because prey visual detection, identification, and motion planning before launching the attack generally occurs at far distances while the predator is in a perch [19], [43].

Differences in head movement strategies while perching could be related to between-species differences in some components of the visual fields reported in an accompanying study [23] or simply reflect visual responses to habitat structure. We measured scanning behavior in the habitats each species generally uses to visually search for prey, so we believe our findings are relevant for the habitat types studied. Additionally, we recorded individuals under captive conditions, and obtained similar results. However, it is possible that some confounding factors may have played a role in our findings. For instance, individuals may have tracked visually the observer with the camera (although in our recordings we tried to be as far away as possible and use the camera zoom). Even though the observer was a visual stimulus, the scanning behavior of focal birds might be different when tracking a prey item. Additionally, we were not able to establish the degree of visual attention to prey searching (focal animals may have been loafing, digesting food, etc.). However, previous studies have used a similar approach to ours (an observer within the visual field of a focal bird) to characterize scanning behavior in other raptors [10]. We expect that the behaviors we recorded represent the main strategies these species employ to scan the environment, but acknowledge that other strategies may be used to visually track specific types of visual stimuli.

Red-tailed Hawks scan from high vantage points (>10 m [44]) in open areas, which enhance the visibility to detect prey [19], reducing visual obstruction effects due to vegetation being farther away from individuals perching. Red-tailed Hawks have large lateral visual fields and comparatively narrow binocular fields [23]. This visual field configuration emphasizes the importance of lateral vision for prey detection, as the foveae subtends the lateral visual fields [45], [46], thereby increasing the distance at which individuals can resolve prey items. One of the shortcomings of having long intervals between consecutive head movements (i.e., fixating on an image longer) is that the visually static background would fade with time, but this effect would actually increase the saliency of any moving object [1], [47]. We propose that the slow regular head movements would be part of a fixation sequence to increase the ability of Red-tailed Hawks to detect cues related to prey movement with their lateral fields, given that the visual acuity of this species is likely higher than that of the prey [19]. An additional effect of slow head movements is the reduction in the chances of predators being spotted by prey, which may be beneficial to reduce anti-predator behavior as Red-tailed Hawks scan from relatively exposed perches.

Interestingly, Bald Eagles (*Haliaeetus leucocephalus*) also have long intervals between consecutive head movements [10]. This suggests that this head movement strategy may be common in large Accipitridae falcons inhabiting open areas. Fite and Rosenfield-Wessels [26] found that the width of the foveal depression is narrower in species with large eyes. The narrow and deep foveal depression with a spherical pit may reduce light scattering [48] and facilitate image magnification [24], image fixation and exaggeration of small movements [49], and directional focus [50]. These foveal properties could increase detection rates of moving prey [22].

Cooper's Hawks occupy closed habitats with substantial visual obstruction due to vegetation [28]. They have narrow blind areas behind their heads [23], which increase the volume of space they can visually cover without head movement. However, Cooper's Hawks had the highest rate of regular head movements, likely to enhance visual tracking of objects in a visually cluttered environment by quickly shifting the image between the right and left central foveae. Cooper's Hawks usually track fast prey that move in three dimensions, whereas Red-tailed Hawks and American Kestrels generally track prey that may move a slightly lower speeds or that move in two dimensions. Black phoebes, a sit-and-wait predator that detects small insects from perches before attacking them, also shows high head movement rates in territories with high tree cover compared with those with low tree cover [15]. Cooper's Hawks also have intermediate rates of translational head movements (this study) and large degrees of eye movement [23]. This suggests that Cooper's Hawks may complement the regular head movements with depth cues from motion parallax provided by translational head movements to establish the relative distance to objects [31].

American Kestrels showed the highest rate of translational head movements compared to the two hawks studied. Translational head movements have been considered the primary generators of depth information through motion parallax [31], [33]. However, recent evidence in monkeys indicate that middle temporal neurons compute depth cues involved in motion parallax based upon the slow eye movements that are part of the optokinetic response [37], [51]. Therefore, both head and eye movements appear to have an important role in generating depth information for motion parallax depending on the distance between the observer and the target of visual attention [36]. Using a opthalmoscopic reflex technique, American Kestrels showed a limited degree of eye movement (∼1°) in relation to the other two hawks studied ([23], but see [52], [53]). We propose that American Kestrels may compensate for this reduced degree of eye movement by increasing the frequency of translational head movements to obtain the necessary depth information from both monocular views. Fox et al. [54] suggested that American Kestrels also uses stereopsis for binocular depth perception, which involves assessing object solidity and depth based on binocular disparity cues [55]. Owls possess both mechanisms of depth perception (motion parallax and stereopsis), a phenomenon called primary-depth-cue-equivalence [38], [56]. It is then possible that American Kestrels use their relatively wide binocular and lateral visual fields for primary-depth-cue-equivalence to locate small and cryptic invertebrate prey items from perches or by hovering above them [23].

In the three studied species, regular head movements were more common than translational head movements. The other raptor species whose head movement patterns have been studied in detail is the Barn Owl, which increases head movements parallel to the direction of prey movement by combining regular and translational movements [33], [43], [57]. According to Ohayon et al. [33], regular and translational head movements were similar in number when Barn Owls detected a prey item before attacking it. Differences in head movement patterns between our studied species and owls may arise as a result of the more frontal placement of the owl eyes [58], their lack of eye movement [59], their retinal structure (i.e., owls have a visual streak and a temporal fovea rather than two foveae [60]). Although these visual traits and head movement strategy allow owls to enhance predator detection, they also pose sensory constraints that can be used by prey to reduce mortality. For instance, in Barn Owls, narrow lateral visual fields and low degree of eye movement may reduce their hunting success when prey move sideways from the line of attack [42], [61]. Consequently, characterizing inter-specific differences in head movement strategies involved in prey detection can have relevant implications for predator-prey interactions.

Our results, along those of an accompanying study on the visual fields of these three diurnal raptors [23], underscore the association between visual field configuration and head movement strategies to scan the environment in different species. We conclude that diurnal raptors have species-specific strategies to gather visual information, likely about prey, while perching. Future studies under controlled conditions (using head cameras [57] or gaze trackers [2]) should ascertain the functional nature of these scanning strategies (e.g., fixation vs. peering).

## Supporting Information

Text S1 Video examples of the characteristic head movement strategies of each species.
(0.03 MB DOC)

Video S1 Head movement patterns of a Cooper's Hawk.
(4.07 MB WMV)

Video S2 Head movement patterns of a Red-tailed Hawk.
(8.28 MB WMV)

Video S3 Head movement patterns of an American Kestrel.
(9.93 MB WMV)

## References

[1] Land MF (1999). Motion and vision: why animals move their eyes.. J Comp Physiol [A].

[2] Kjærsgaard A, Pertoldi C, Loeschcke V, Hansen DW (2008). Tracking the gaze of birds.. J Avian Biol.

[3] Kaminski J, Riedel J, Call J, Tomasello M (2005). Domestic goats, *Capra hircus*, follow gaze direction and use social cues in an object choice task.. Anim Behav.

[4] Tomonaga M, Imura T (2010). Visual search for human gaze direction by a chimpanzee (*Pan troglodytes*).. Plos ONE.

[5] Wolfe J, De Valois KK (2000). Visual attention.. Seeing. 2nd ed.

[6] Bradbury JW, Vehrencamp SL (1998). Principles of animal communication..

[7] Dukas R (2004). Causes and consequences of limited attention.. Brain Behavior and Evolution.

[8] Fernández-Juricic E, Erichsen JT, Kacelnik A (2004). Visual perception and social foraging in birds.. Trends Ecol Evol.

[9] Treves A (2000). Theory and method in studies of vigilance and aggregation.. Anim Behav.

[10] Tucker VA (2000). The deep fovea, sideways vision and spiral flight paths in raptors.. J Exp Biol.

[11] Dawkins MS (2002). What are birds looking at? Head movements and eye use in chickens.. Anim Behav.

[12] Gall MD, Fernández-Juricic E (2010). Visual fields, eye movements, and scanning behavior of a sit-and-wait predator, the Black Phoebe (*Sayornis nigricans*).. J Comp Physiol A.

[13] Jones KA, Krebs JR, Whittingham MJ (2007). Vigilance in the third dimension: head movement not scan duration varies in response to different predator models.. Anim Behav.

[14] Land MF (1999b). The roles of head movements in the search and capture strategy of a tern.. J Comp Physiol [A].

[15] Gall MD, Fernández-Juricic E (2009). Effects of physical and visual access to prey on patch selection and food search effort in a sit-and-wait predator, the Black Phoebe.. Condor.

[16] Griesser M (2003). Nepotistic vigilance behavior in Siberian jay parents.. Behav Ecol.

[17] Mouritsen H, Feenders G, Liedvogel M, Kropp W (2004). Migratory birds use head scans to detect the direction of the earth's magnetic field.. Curr Biol.

[18] Fujita M (2004). Kinematic parameters of the walking of herons, ground-feeders, and waterfowl.. Comp Biochem Physiol A.

[19] Andersson M, Wallander J, Isaksson D (2009). Predator perches: a visual search perspective.. Funct Ecol.

[20] Andersson M (1981). On optimal predator search.. Theor Popul Biol.

[21] Stevens DW, Krebs JR (1986). Foraging theory..

[22] Jones MP, Pierce KE, Ward D (2007). Avian vision: A review of form and function with special consideration to birds of prey.. J Exotic Pet Med.

[23] O'Rourke CT, Hall MI, Pitlik T, Fernández-Juricic E (2010). Hawk eyes I: diurnal raptors differ in visual fields and degree of eye movement.. PLoS ONE.

[24] Walls GL (1942). The vertebrate eye and its adaptive radiation.. Bull no. 19.

[25] Meyer DB, Crescitelli F (1977). The avian eye and its adaptations.. The visual system in vertebrates, Handbook of Sensory Physiology, vol. VII/5.

[26] Fite KV, Rosenfield-Wessels S (1975). A comparative study of deep avian foveas.. Brain Behav Evol.

[27] Preston CR, Beane RD (2009). Red-tailed Hawk (*Buteo jamaicensis*). In: Poole A, editor. The birds of North America online. Ithaca: Cornell Lab of Ornithology.. http://bna.birds.cornell.edu/bna/species/052.

[28] Curtis OE, Rosenfield RN, Bielefeldt J (2006). Cooper's Hawk (*Accipiter cooperii*). In: Poole A, editor. The birds of North America online. Ithaca: Cornell Lab of Ornithology.. http://bna.birds.cornell.edu/bna/species/075.

[29] Smallwood JA, Bird DM (2002). American Kestrel (*Falco sparverius*). In: Poole A, editor. The birds of North America online. Ithaca: Cornell Lab of Ornithology.. http://bna.birds.cornell.edu/bna/species/602.

[30] Kral K (1998). Side-to-side head movements to obtain motion depth cues: a short review of research on the praying mantis.. Behav Process.

[31] Kral K (2003). Perspectives on the role of head movements in depth perception.. Behav Process.

[32] Gibson JJ (1950). The perception of the visual world..

[33] Ohayon S, van der Willigen RF, Wagner H, Katsman I, Rivlin E (2006). On the barn owls visual pre-attack behavior: I. structure of head movements and motion patterns.. J Comp Physiol [A].

[34] Casperon LW (1999). Head movements and vision in underwater-feeding birds of stream, lake and seashore.. Bird Behav.

[35] Nawrot M (2003). Eye movements provide the extra-retinal signal required for the perception of depth from motion parallax.. Vision Research.

[36] Nawrot M (2003). Depth from motion parallax scales with eye movement gain.. Journal of Vision.

[37] Nadler JW, Angelaki DE, DeAngelis GC (2008). A neural representation of depth from motion parallax in macaque visual cortex.. Nature.

[38] van der Willigen RF, Frost BJ, Wagner H (2002). Depth generalization from stereo to motion parallax in the owl.. J Comp Physiol [A].

[39] Blumstein DT, Daniel JC (2007). Quantifying behavior the JWatcher way..

[40] Wallman J, Letelier JC, Zeigler HP, Bischof HJ (1993). Eye movements, head movements and gaze stabilization in birds.. Vision, brain and behaviour in birds.

[41] Tucker VA (2000). Gliding flight: Drag and torque of a hawk and a falcon with straight and turned heads, and a lower value for the parasite drag coefficient.. J Exp Biol.

[42] Shifferman E, Eilam D (2004). Movement and direction of movement of a simulated prey affect the success rate in barn owl *Tyto alba* attack.. J Avian Biol.

[43] Fux M, Eilam D (2009). How barn owls (*Tyto alba*) visually follow moving voles (*Microtus socialis*) before attacking them.. Physiol Behav.

[44] Leyhe JE, Ritchison G (2004). Perch sites and hunting behavior of Red-tailed Hawks (*Buteo jamaicensis*).. J Raptor Res.

[45] Reymond L (1985). Spatial visual acuity of the eagle *Aquila audax*: a behavioural, optical and anatomical investigation.. Vision Res.

[46] Reymond L (1987). Spatial visual acuity of the Falcon, *Falco berigora*: a behavioural, optical and anatomical investigation.. Vision Res.

[47] Kelly DH (1979). Motion and vision. II. stabilized spatio-temporal threshold surface.. J Opt Soc Am.

[48] Martin GR (1986). Shortcomings of an eagle's eye.. Nature.

[49] Pumphrey RF (1948). The theory of the fovea.. J Exp Biol.

[50] Harkness L, Bennet-Clark HC (1978). The deep fovea as a focus indicator.. Nature.

[51] Nadler JW, Nawrot M, Angelaki DE, DeAngelis GC (2009). MT neurons combine visual motion with a smooth eye movement signal to code depth-sign from motion parallax.. Neuron.

[52] Pettigrew JD, Cool S, Smith EL (1978). Comparison of the retinotopic organization of the visual wulst in noctural and diurnal raptors, with a note on the evolution of of frontal vision.. Frontiers in visual science.

[53] Frost BJ, Wise LZ, Morgan B, Bird D (1990). Retinotopic representation of the bifoveate eye of the kestrel (*Falco sparverius*) on the optic tectum.. Visual Neurosci.

[54] Fox R, Lehmkuhle SW, Bush RC (1977). Stereopsis in the falcon.. Science.

[55] Poggio G, Poggio T (1984). The Analysis of Stereopsis.. Annu Rev of Neurosci.

[56] van der Willigen RF, Frost BJ, Wagner H (1998). Stereoscopic depth perception in the owl.. Neuroreport.

[57] Ohayon S, Harmening W, Wagner H, Rivlin E (2008). Through a barn owl's eyes: interactions between scene content and visual attention.. Biol Cybern.

[58] Martin GR (1986). Sensory capacities and the nocturnal habit in owls (Strigiformes).. Ibis.

[59] Steinbach MJ, Money KE (1973). Eye movements of the owl.. Vision Res.

[60] Wathey J, Pettigrew J (1989). Quantitative analysis of the retinal ganglion cell layer and optic nerve of the barn owl *Tyto alba*.. Brain Behav Evol.

[61] Ilany A, Eilam D (2008). Wait before running for your life: defensive tactics of spiny mice (*Acomys cahirinus*) in evading barn owl (*Tyto alba*) attack.. Behav Ecol Sociobiol.

