# Supplementary material for: Hawk Eyes II: Diurnal Raptors Differ in Head Movement Strategies When Scanning from Perches
Source: PLoS One. 2010 Sep 22;5(9):e12169. doi: 10.1371/journal.pone.0012169 (PMC2943910; doi:10.1371/journal.pone.0012169)
Supplement: Text S1 — Video examples of the characteristic head movement strategies of each species. (0.03 MB DOC) [file pone.0012169.s001.doc]

Supporting information S1 for manuscript entitled “Hawk eyes II: diurnal raptors differ in head movement strategies when scanning from perches”

Videos of the patterns of head movements of each of this species recorded in the field are available at the Macaulay Library Sound and Video Catalog ([http://animalbehaviorarchive.org](http://animalbehaviorarchive.org/)). Here are some examples:

Cooper's Hawk: <http://macaulaylibrary.org/video/8079>

Red-tailed Hawk: <http://macaulaylibrary.org/video/51688>

American Kestrel: <http://macaulaylibrary.org/video/2032>

Videos recorded in captive conditions of a Cooper’s Hawk (Video S2), a Red-tailed Hawk (Video S3), and an American Kestrel (Video S4) showing examples of the patterns of head movements characteristic of each species.

Comparatively, Cooper’s Hawks have high rates of regular head movements, Red-tailed Hawks have long intervals between consecutive regular head movements, and American Kestrels have high rates of translational head movements.
